# Supplementary material for: A cascading nonlinear magneto-optical effect in topological insulators
Source: Sci Rep. 2018 Mar 2;8:3908. doi: 10.1038/s41598-018-22196-x (PMC5834634; doi:10.1038/s41598-018-22196-x)
Supplement: Supplementary file 1 — Supplementary Information [file 41598_2018_22196_MOESM1_ESM.pdf]

Supplementary Information

A cascading nonlinear magneto-optical effect in topological insulators

Richarj Mondal[1], Yuta Saito[2], Yuki Aihara[1], Paul Fons[2], Alexander V. Kolobov[2], Junji Tominaga[2] , Shuichi Murakami[3] & Muneaki Hase[1,2,⋆]

[1]*Division of Applied Physics, Faculty of Pure and Applied Sciences, University of Tsukuba, 1-1-1 Tennodai, Tsukuba 305-8573, Japan.*

[2]*Nanoelectronics Research Institute, National Institute of Advanced Industrial Science and Technology, Tsukuba Central 5, 1-1-1 Higashi, Tsukuba 305-8565, Japan.*

[3]*Department of Physics, Tokyo Institute of Technology, 2-12-1 Ookayama, Meguro-ku, Tokyo 152-8551, Japan.*

⋆e-mail: mhase@bk.tsukuba.ac.jp

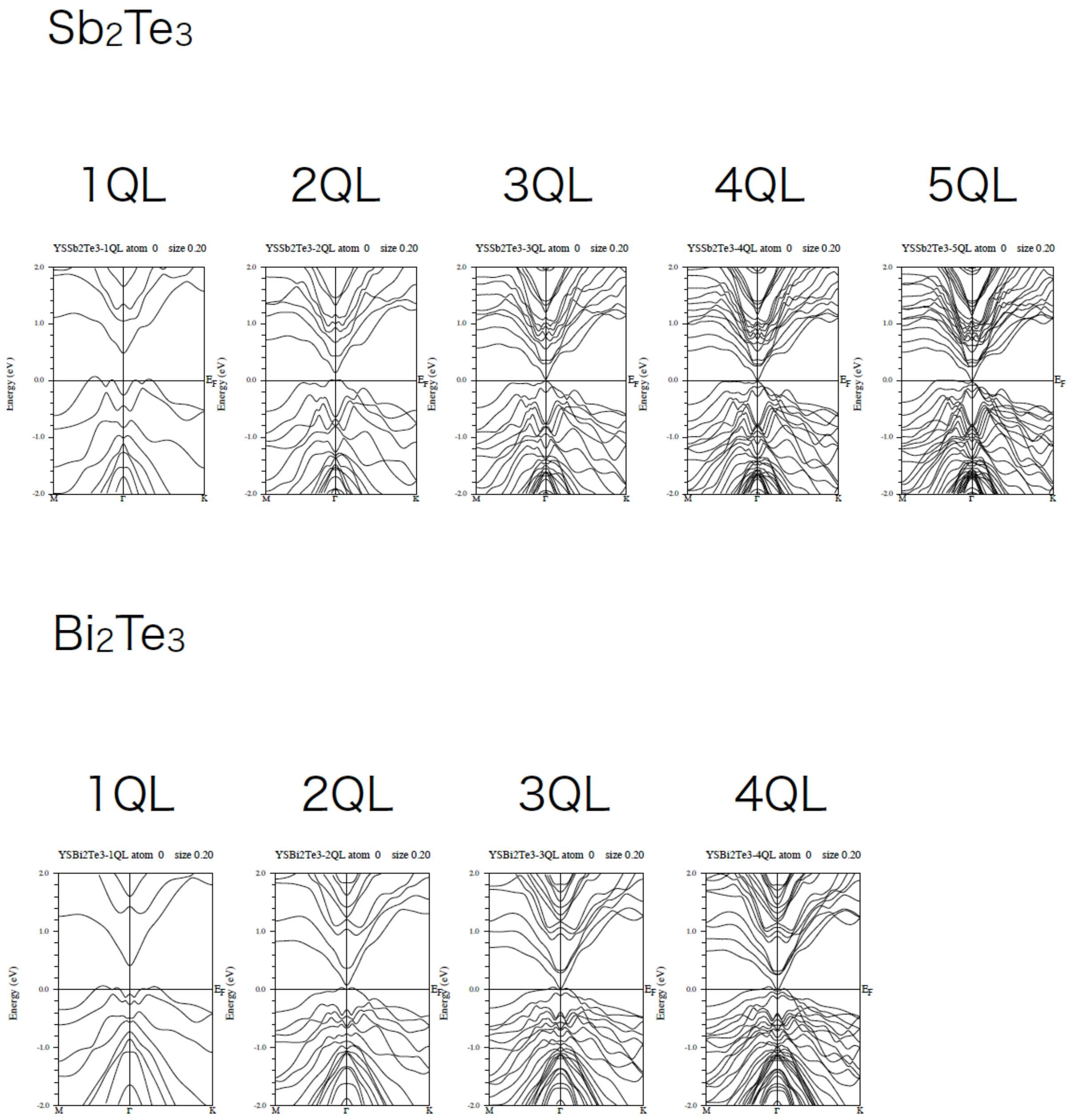

Figure S1. Band structures of ultrathin Sb_2Te_3 and Bi_2Te_3 calculated by using first-principles density functional theory.
